# The role of the liver X receptor in chronic obstructive pulmonary disease

**DOI:** 10.1186/1465-9921-14-106

**Published:** 2013-10-12

**Authors:** Andrew Higham, Simon Lea, Jonathan Plumb, Barbara Maschera, Karen Simpson, David Ray, Dave Singh

**Affiliations:** 1 The University of Manchester, NIHR Translational Research Facility, University Hospital of South Manchester Foundation Trust, Southmoor Road, Manchester M23 9LT, UK; 2 GlaxoSmithKline, Respiratory CEDD, Gunnels Wood Road, Stevenage, Hertfordshire SG1 2NY, UK

**Keywords:** COPD, Liver X receptor, Alveolar macrophage, Inflammatory cytokines

## Abstract

**Background:**

There is a need for novel anti-inflammatory therapies to treat COPD. The liver X receptor (LXR) is a nuclear hormone receptor with anti-inflammatory properties.

**Methods:**

We investigated LXR gene and protein expression levels in alveolar macrophages and whole lung tissue from COPD patients and controls, the effect of LXR activation on the suppression of inflammatory mediators from LPS stimulated COPD alveolar macrophages, and the effect of LXR activation on the induction of genes associated with alternative macrophage polarisation.

**Results:**

The levels of LXR mRNA were significantly increased in whole lung tissue extracts in COPD patients and smokers compared to non-smokers. The expression of LXR protein was significantly increased in small airway epithelium and alveolar epithelium in COPD patients compared to controls. No differences in LXR mRNA and protein levels were observed in alveolar macrophages between patient groups. The LXR agonist GW3965 significantly induced the expression of the LXR dependent genes ABCA1 and ABCG1 in alveolar macrophage cultures. In LPS stimulated alveolar macrophages, GW3965 suppressed the production of CXCL10 and CCL5, whilst stimulating IL-10 production.

**Conclusions:**

GW3965 did not significantly suppress the production of TNFα, IL-1β, or CXCL8. Our major finding is that LXR activation has anti-inflammatory effects on CXC10, CCL5 and IL-10 production from alveolar macrophages.

## Introduction

Cigarette smoking causes oxidative stress and inflammation in the airways [1], and is a major risk factor for the development of chronic obstructive pulmonary disease (COPD). This condition is characterised by progressive airway inflammation [2] involving a complex network of inflammatory cells. The number of lung macrophages is increased in COPD [3], and these cells are thought to play a key role in inflammation and tissue destruction in COPD [4]. There is a need for novel anti-inflammatory drugs to treat airway inflammation in COPD.

Liver X receptor (LXR) is a nuclear hormone receptor that exists in two isoforms; LXRα and LXRβ [5]. LXR is a sensor of cellular cholesterol load [6], and regulates the transcription of genes involved in cholesterol efflux [7-9] and low density lipoprotein receptor degradation [10]. Consequently, there has been much interest in the potential of LXR agonists for the treatment of atherosclerosis [11], and it has been demonstrated that these drugs reduce plaque size in animal models [12]. There is also evidence that LXR activation results in anti-inflammatory effects; LXR agonists reduce the expression of inflammatory genes in animal models [13], suppress the expression of a subset of LPS-induced inflammatory genes in mouse macrophages [14] and inhibit cytokine production from lymphocytes [15]. LXR exerts these anti-inflammatory effects by preventing co-repressor removal from the promoter regions of targeted genes, thereby suppressing transcription [16,17].

Birrell et al. [18] demonstrated that the LXR agonist GW3965 decreased LPS-induced airway neutrophilia in rats. Furthermore, LXR gene expression was detectable in human alveolar macrophages, and GW3965 caused up to 60% inhibition of cytokine production from these cells. These findings suggest that LXR agonists may have the potential to reduce airway inflammation in COPD through the modulation of macrophage function. To further investigate this possibility, the findings of Birrell et al. need to be confirmed using COPD alveolar macrophages. COPD alveolar macrophages are phenotypically different from healthy controls [19], and the effects of LXR activation on cytokine production may therefore be altered. Furthermore, it is not known whether LXR expression is changed within the lungs of COPD patients compared to controls.

ATP-binding cassette (ABC) A1 is an LXR dependent gene that is involved in cholesterol efflux [20]. ABCA1 appears to play a role in the polarisation of macrophages away from the classical pro-inflammatory phenotype (M1), towards the alternative phenotype (M2) that can exert anti-inflammatory and tissue repair effects [21]. It is possible that LXR mediated skewing of lung macrophages towards an alternative activation phenotype may be therapeutically beneficial in COPD.

In order to further understand the potential of LXR agonists as anti-inflammatory drugs in COPD, we have investigated the expression and function of LXR in COPD pulmonary cells, focusing on alveolar macrophages. We studied the effects of LXR activation on cytokine production from COPD compared to control alveolar macrophages and evaluated possible effects on alternative macrophage activation. We investigated whether LXR expression was changed in COPD compared to control lungs; we observed, as expected, LXR expression in alveolar macrophages, but also demonstrated expression in airway epithelium and in lymphocytes. We therefore also studied the effects of LXR activation on cytokine production from bronchial epithelial cells and peripheral blood mononuclear cells (PBMCs).

## Methods

### Subjects

109 patients undergoing surgical resection for lung cancer were recruited (demographics shown in Table 1). 10 COPD patients and 10 healthy non-smokers were also recruited to donate peripheral blood (demographics shown in Additional file 1). COPD patients had ≥ 10 pack years smoking history, typical symptoms and airflow obstruction. Controls were either smokers (S) with normal lung function or lifelong non-smokers (NS). Immunohistochemistry and gene expression experiments used historically collected samples allowing control for smoking status; current smoking COPD patients and S were compared to NS. Cell culture experiments required fresh cells which had limited availability; we therefore recruited ex-smokers and current smokers, both with and without COPD, for these experiments. All subjects gave written informed consent. This research was approved by the local research ethics committee.

**Table 1:** Subject demographics for patients recruited undergoing lung resection

	**NS**	**S**	**COPD**
**n**	23	41	45
**Gold stage I**	n/a	n/a	9
**Gold stage II**	n/a	n/a	33
**Gold stage III**	n/a	n/a	3
**Age (yrs)**	64.5 (11.4)	63 (12.1)	64.2 (7)
**Sex (M/F)**	7/16	20/21	19/26
**FEV**_**1 **_**(L)**	2.3 (0.7)	2.4 (0.8)	1.7 (0.5)
**FEV**_**1 **_**% predicted**	102 (23.9)	92.4 (14.1)	68.3 (12.8)
**FVC (L)**	3 (0.8)	3.3 (0.9)	3.0 (0.9)
**FEV**_**1**_**/FVC ratio (%)**	73.3 (9)	73.4 (7.9)	58.9 (11.8)
**Pack year history**	0	41.7 (22.1)	51.5 (23)
**Current smoker (%)**	0	70	89
**ICS users**	0	0	12

Data shown are mean (sd). NS: non-smokers, S: smokers, FEV_1_: forced expiratory volume in 1s, FVC: forced vital capacity, ICS: inhaled corticosteroid.

### Tissue sampling and processing

Tissue blocks were labelled with LXRα or LXRβ, as described in Additional file 2.

Details of lung tissue RNA extraction are in Additional file 2.

### Alveolar macrophage isolation and culture

Alveolar macrophages were isolated from lungs as previously described [22]. Macrophages were analysed for LXRα and LXRβ mRNA expression (described in Additional file 2).

#### LXR dependent genes

Macrophages were cultured with GW3965 (1 μM) (Sigma-Aldrich) or vehicle control (DMSO 0.05%, now referred to as “vehicle”) for 4, 24, or 48 h. RNA extraction and real time PCR analysis for expression levels of ABCA1, ABCG1, toll-like receptor 4 (TLR4), hemeoxygenase 1 (HO-1), cluster of differentiation (CD) 36, and mannose receptor (MR) is described in Additional file 2.

#### Inflammatory mediator production

Macrophages were cultured with GW3965 (1 or 10 μM), dexamethasone (1 μM) (Sigma-Aldrich), or vehicle for 1 h followed by LPS (1 μg/ml, *Escherichia Coli* B6-026, Sigma-Aldrich) stimulation and inflammatory mediator production was analysed at 6 and 24 h (described in Additional file 2).

#### STAT1 phosphorylation

Macrophages were cultured with GW3965 (1 or 10 μM) or vehicle for 1 h followed by LPS stimulation for 1 h. Protein was extracted and analysed by Western Blot (described in Additional file 2).

### PBMC culture

Full details are in Additional file 2; PBMCs were isolated and cultured with GW3965 (1 or 10 μM), dexamethasone (1 μM), or vehicle for 1 h followed by stimulation with anti-CD2/3/28 antibody (24 h) and measurement of supernatant cytokines.

### Epithelial cell culture

Full details are in Additional file 2; BEAS-2B cells, a human bronchial epithelial cell line, (American Type Culture Collection, Middlesex, UK) were cultured and labelled with LXRα or LXRβ antibodies. Cells were also cultured with GW3965 (1 or 10 μM), dexamethasone (1 μM), or vehicle for 1 h followed by LPS (1 μg/ml) [23] or poly I:C (10 μg/ml) (Invivogen, San Diego, California) [24] stimulation for 24 h. Supernatants were analysed for CXCL10 by ELISA.

### Data analysis

Normally distributed data were compared using a repeated measures ANOVA followed by a paired t-test or a one-way ANOVA followed by an unpaired t-test. Non-normally distributed data were compared using a Friedman test followed by a Wilcoxon matched pairs test or a Kruskal-Wallis followed by a Mann–Whitney test. P<0.05 was considered significant.

## Results

### LXR mRNA expression

**Figure 1:**
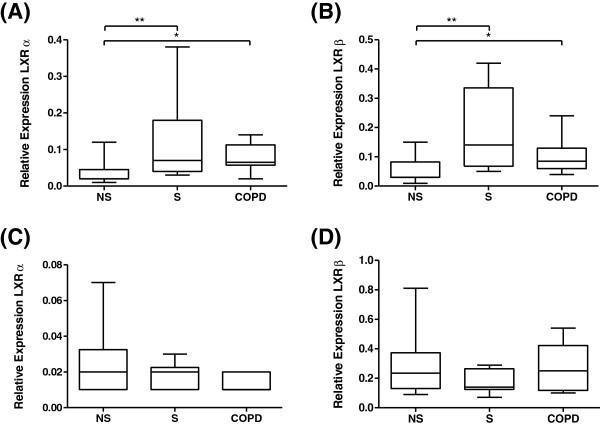
**LXRα and LXRβ mRNA expression in whole lung tissue and lung macrophages.** The expression of LXRα mRNA **(A and C)** and LXRβ mRNA **(B and D)** in whole lung tissue **(A and B)** and lung macrophages **(C and D)** from 10 non-smoking controls (NS), 10 smoking controls (S) and 10 COPD patients. Data shown are median ± range of relative LXRα/β expression levels. *, ** = Significantly increased expression above NS (p<0.05, p<0.01 respectively).

LXRα and LXRβ mRNA expression levels were analysed within the whole lung tissue of 10 NS, 10 S, and 10 COPD patients. LXRα and LXRβ mRNA expression levels were significantly increased in COPD patients and S compared to NS (Figure 1). LXRα and LXRβ mRNA expression levels in whole lung tissue were similar in COPD patients compared to S (p=0.82 and p=0.16 respectively). In macrophages there was no difference in the mRNA levels between the 3 groups of patients (Figure 1).

### LXR protein expression

**Figure 2:**
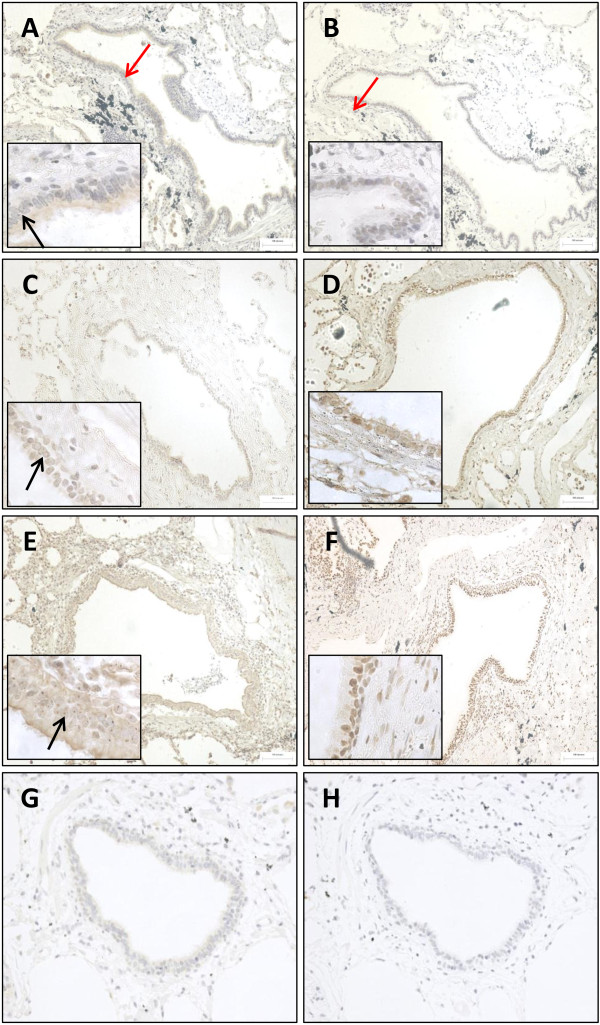
**Representative images of LXRα and LXRβ expression in small airway epithelium and subepithelium.** The distribution of LXRα **(A, C, and E)** and LXRβ **(B, D, and F)** in the epithelium (red arrow in **A**) and subepithelium (red arrow in **B**) of small airways, present in lung sections of non-smoking controls (NS) **(A-B)**, smoking controls (S) **(C-D)** and COPD patients **(E-F)**. LXRα and LXRβ were detected using 3,3’-diaminobenzidine (brown; positive LXRα stain indicated by black arrow) and cell nuclei were counterstained using Meyer’s haematoxylin. Substitution of LXRα **(G)** and LXRβ **(H)** primary antibodies for isotype controls displayed no immunoreactivity.

**Figure 3:**
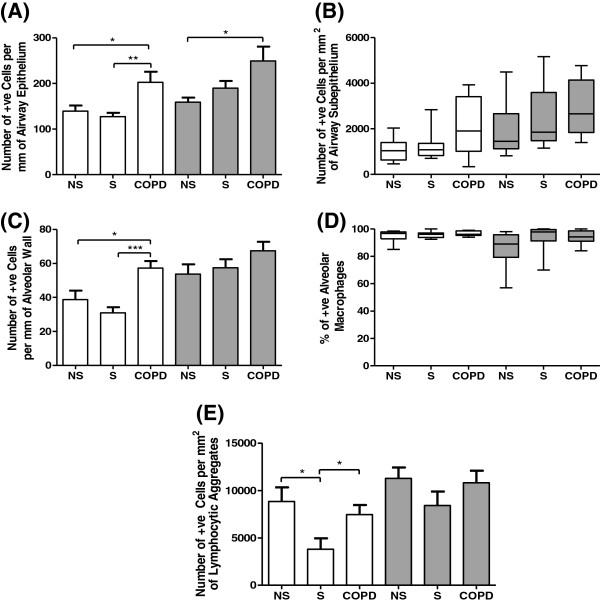
**Quantification of LXR protein in lung tissue sections.** The distribution of LXRα (white bars) and LXRβ (grey bars) was quantified in the epithelium **(A)** and subepithelium **(B)** of small airways, the alveolar wall **(C)**, in alveolar macrophages **(D)**, and within lymphocytic aggregates **(E)**. Data shown are mean ± SEM **(A, C, and E)** or median ± range **(B and D)** of 10 non-smoking controls (NS) (apart from E n=8), 10 smoking controls (S) (apart from E n=6) and 10 COPD patients (apart from E n=9). *, ** and *** = significant difference in protein levels (p<0.05, p<0.01 and p<0.001 respectively).

LXR protein expression was analysed within formalin fixed paraffin embedded lung tissue sections of 10 NS, 10 S, and 10 COPD patients. The number of LXRα immunoreactive cells was significantly increased in the small airways epithelium of COPD patients compared to NS (p=0.03) and S (p=0.007) (Figures 2 and 3). The number of cells expressing LXRβ was significantly increased in the small airways epithelium of COPD patients compared to NS (p=0.01).

**Figure 4:**
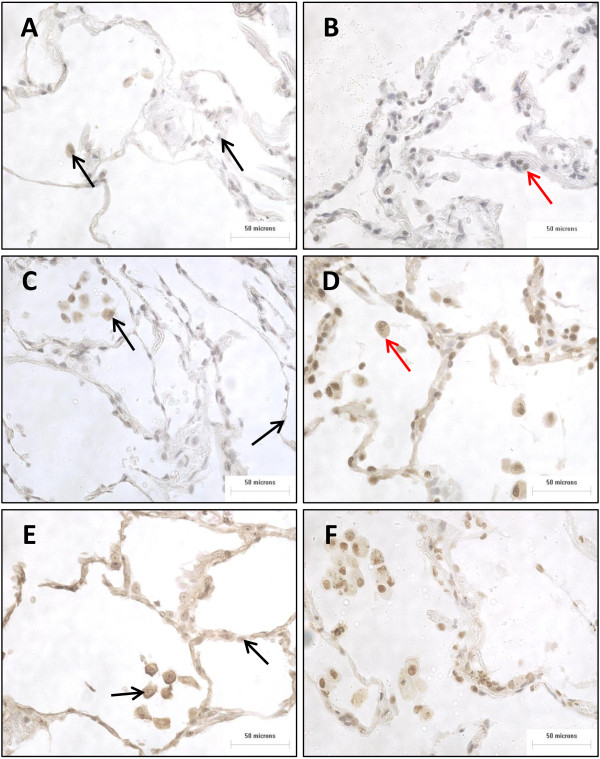
**Representative images of LXRα and LXRβ expression in alveolar epithelium and alveolar macrophages.** The distribution of LXRα **(A, C, and E)** and LXRβ **(B, D, and F)** in the alveolar wall (red arrow in **B**) and in alveolar macrophages (red arrow in **D**) present in lung sections of NS **(A-B)**, S **(C-D)** and COPD patients **(E-F)**. Positive LXRα stain indicated by black arrow.

The number of LXRα immunoreactive alveolar epithelial cells was significantly increased in COPD patients compared to both NS and S (p=0.01 and p<0.0001 respectively) (Figures 4 and 3). There was no significant difference in the number of LXRβ immunoreactive alveolar epithelial cells between patient groups.

There were numerically increased numbers of cells staining positive for LXRα and LXRβ expression in the subepithelium of small airways of COPD patients, but the differences between groups did not reach statistical significance (Figures 2 and 3). There were no significant differences between groups in the number of LXRα and LXRβ immunoreactive alveolar macrophages.

LXR was expressed within lymphocyte aggregations, which are either organised tertiary lymphoid follicles or lymphocyte clusters without an organised structure [25]. Lymphocyte aggregations were observed in 8 NS, 6 S, and 9 COPD patients (out of a possible n=10 per group). The number of LXRα immunoreactive cells was significantly increased within the lymphocytic aggregates in NS and COPD patients compared to S (p=0.03 for both groups; Additional file 3). There was no difference between NS and COPD patients. The number of LXRβ immunoreactive cells within lymphocytic aggregates was similar in the three groups.

### Effect of GW3965 on alveolar macrophages

#### LXR dependent genes

Macrophages from 8 S and 8 COPD patients were treated with the LXR agonist GW3965 and ABCA1 and ABCG1 mRNA expression was quantified. GW3965 significantly increased the gene expression levels of the LXR dependent genes ABCA1 and ABCG1; see Additional file 4.

#### Protein secretion

The effect of GW3965 on LPS stimulated inflammatory mediator production was investigated in macrophages from 8 S and 7 COPD patients. LPS significantly increased cytokine production from COPD and S macrophages, with the increase in CXCL10 in COPD patients failing to reach statistical significance (p=0.09) (Additional file 5).

**Figure 5:**
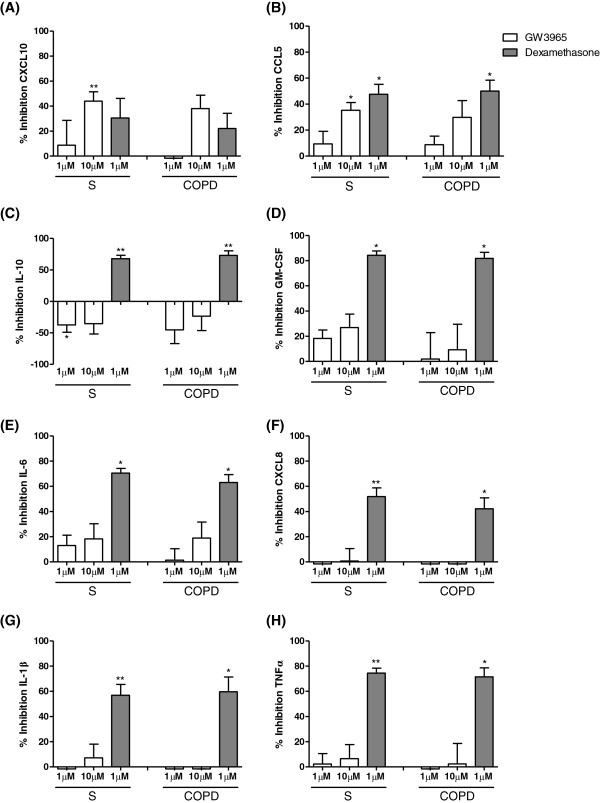
**The effect of GW3965 on LPS stimulated inflammatory mediator production in alveolar macrophages from COPD patients and S compared.** Results are presented separately for macrophages from smoking controls (n=8) and COPD patients (n=7) that were treated with GW3965 (1 μM or 10 μM) (white bars), or dexamethasone (1 μM) (dark grey bars) for 1 h prior to stimulation with LPS (1 μg/ml) for 24 h. Culture supernatants were analysed for CXCL10 **(A)**, CCL5 **(B)**, IL-10 **(C)**, GM-CSF **(D)**, IL-6 **(E)**, CXCL8 **(F)**, IL-1β **(G)**, and TNFα **(H)**. Data shown are mean ± SEM. * and ** = significant mediator reduction below vehicle control calculated using absolute values (p<0.05 and p<0.01 respectively).

GW3965 (10 μM) significantly inhibited the production of CXCL10 and CCL5 from S macrophages by 44% and 35% respectively (p=0.006 and p=0.02 respectively; see Figure 5). GW3965 (10 μM) reduced the production of CXCL10 and CCL5 from COPD macrophages by 38% and 30% respectively, but these changes did not reach statistical significance (p=0.1 and p=0.2 respectively). GW3965 did not significantly alter the production of GM-CSF, TNFα, IL-1β, CXCL8 or IL-6 from either subject group (Figure 5). GW3965 (1 μM) significantly increased IL-10 release from S by 37% and caused a non-significant increase from COPD macrophages by 45% (p=0.3). Dexamethasone significantly inhibited the production of all cytokines from S and COPD patients apart from CXCL10 (p=0.06 and p=0.1 respectively). The effects of dexamethasone were similar in COPD patients and S (p>0.05 for each cytokine).

**Figure 6:**
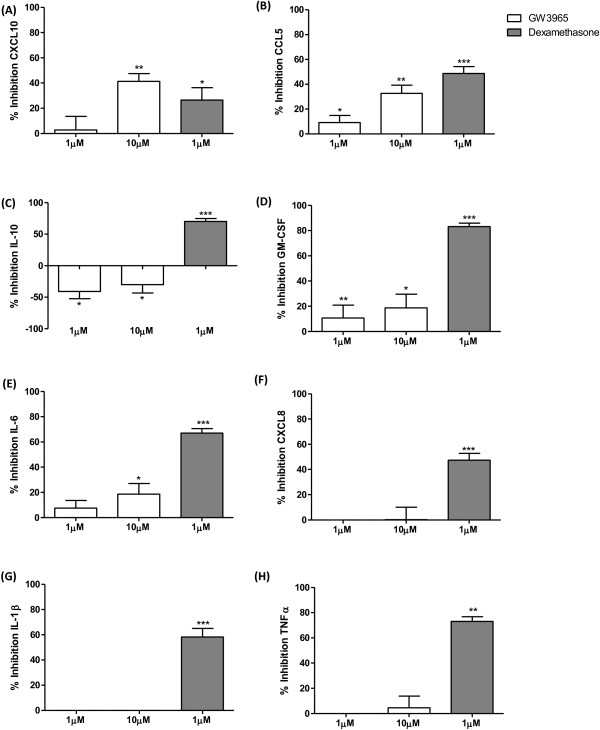
**The effect of GW3965 on LPS stimulated inflammatory mediator production in alveolar macrophages; pooled analysis.** Results are combined for macrophages from smoking controls (n=8) and COPD patients (n=7) that were treated with GW3965 (1 μM or 10 μM) (white bars), or dexamethasone (1 μM) (dark grey bars) for 1 h prior to stimulation with LPS (1 μg/ml) for 24 h. Culture supernatants were analysed for CXCL10 **(A)**, CCL5 **(B)**, IL-10 **(C)**, GM-CSF **(D)**, IL-6 **(E)**, CXCL8 **(F)**, IL-1β **(G)**, and TNFα **(H)**. Data shown are mean ± SEM of combined results. *, ** and *** = significant mediator reduction below vehicle control calculated using absolute values (p<0.05, p<0.01 and p<0.001 respectively).

The effects of GW3965 on CXCL10, CCL5 and IL-10 were numerically similar in COPD patients and S, but were not statistically significant in COPD patients; this may have been due to variability in the limited sample size available from lung surgical resections. We therefore pooled the data from the two groups to increase the power of the statistical analysis (see Figure 6); this pooled analysis showed significant inhibition of CXCL10 and CCL5 as previously noted, but also significant inhibition of GM-CSF and IL-6, (19% and 19% inhibition for both cytokines at 10 μM). The significant increase in IL-10 production was observed again. Dexamethasone significantly inhibited the production of all cytokines.

#### Gene expression

**Figure 7:**
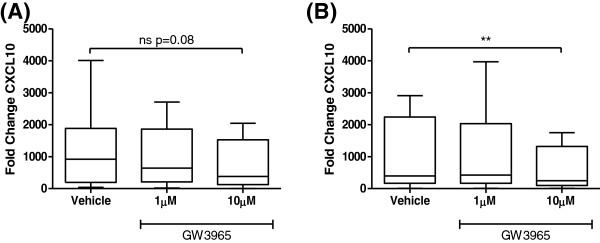
**The effect of GW3965 on CXCL10 mRNA expression in LPS stimulated alveolar macrophages.** Macrophages from smoking controls **(A)** (n=8) and COPD patients **(B)** (n=8) were treated with vehicle (DMSO 0.05%) or GW3965 (1 μM or 10 μM) for 1 h prior to LPS (1 μg/ml) stimulation for 6 h. Data shown are median ± range of fold change. ** = significant difference compared to time matched control (p<0.01 respectively). ns=non-significant.

Having demonstrated that GW3965 inhibited LPS-induced CXCL10 protein secretion from alveolar macrophages, we investigated the effect of GW3965 on CXCL10 mRNA expression levels in macrophages from 8 S and 8 COPD patients in order to understand if this effect was also observed at the level of gene transcription. LPS increased CXCL10 mRNA expression at 6 h but not 24 h (Additional file 6). The effect of GW3965 (10 μM) on CXCL10 mRNA production was therefore studied at 6 h; LPS-induced expression of CXCL10 mRNA was inhibited by 40% from S and 38% from COPD patients (p=0.08 and p=0.008 respectively) (Figure 7).

### The effect of LXR activation on STAT1 phosphorylation

CXCL10 expression is regulated by STAT1 in response to LPS [26] and LXR activation has previously been shown to reduce STAT1 phosphorylation in the human macrophage THP-1 cell line [27]. We therefore studied the effect of GW3965 on STAT1 phosphorylation in LPS stimulated macrophages from 3 S and 3 COPD patients. STAT1 is activated by phosphorylation at two sites; tyrosine 701 and serine 727. LPS stimulation predominantly induces phosphorylation at serine 727 [28].

LPS treatment of S macrophages significantly induced the phosphorylation of STAT1 (727) (p=0.003). LPS treatment of macrophages from COPD patients induced the phosphorylation of STAT1 (727), but this did not reach statistical significance (p=0.07). GW3965 did not significantly inhibit the phosphorylation of STAT1 (727) in S or COPD macrophages even at the highest concentration (10 μM) (p=0.3 for both groups; Additional file 7).

### The effect of LXR activation on macrophage polarisation

We earlier showed that GW3695 upregulated the gene expression levels of the LXR target genes ABCA1 and ABCG1. Now, we observed that GW3965 (1 μM and 10 μM) did not change the expression levels of the known M2 associated genes HO-1, CD36, and MR in S and COPD macrophages (n=8 for both groups) after culture for 4, 24 and 48 h (Additional file 8). In contrast to a previous report [29] we found there was no significant induction of TLR4 gene expression in response to LXR activation in both COPD and S macrophages (Additional file 8).

### PBMC culture

As we had found LXRα and LXRβ immunostaining in lymphoid aggregates of COPD patients and controls (Additional file 3), we decided to investigate the functional effects of LXR activation on cytokine production from peripheral blood lymphocytes of 10 NS and 10 COPD patients. The lymphocytes within PBMCs were activated with anti-CD2/3/28 antibodies, thus significantly increasing cytokine production from NS and COPD patients (Additional file 9). There were no differences in the basal or stimulated cytokine levels between groups.

**Figure 8:**
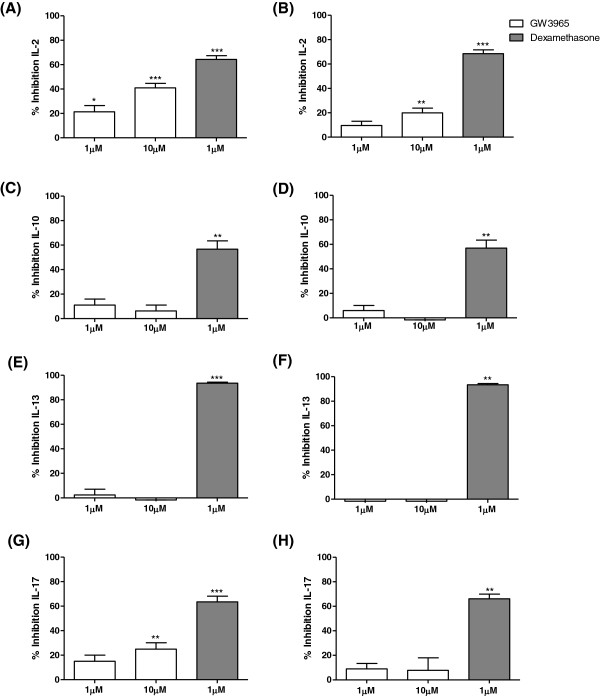
**The effect of GW3965 on anti- CD2/3/28 stimulated inflammatory mediator production from PBMCs.** PBMCs isolated from the peripheral blood of non-smoking controls **(A, C, E, and G)** and COPD patients **(B, D, F, and H)** (n=10 for both groups) were treated with GW3965 (1 μM or 10 μM) (white bars), or dexamethasone (1 μM) (dark grey bars) for 1 h prior to anti-CD2/3/28 stimulation for 24 h. Culture supernatants were analysed for IL-2 **(A-B)**, IL-10 **(C-D)**, IL-13 **(E-F)**, and IL-17 **(G-H)**. Data shown are mean ± SEM. *, **, and *** = significant mediator reduction below vehicle control calculated using absolute values (p<0.05, p<0.01, and p<0.001 respectively).

GW3965 significantly reduced IL-2 and IL-17 release from NS, with 41% and 25% inhibition respectively at 10 μM (p<0.0001, and p=0.01 respectively; Figure 8). There was no effect on IL-10 or IL-13 release. GW3965 (10 μM) significantly inhibited IL-2 production from COPD patients by 20% but had no effect on IL-17, IL-10 or IL-13 release. The effect of GW3965 (10 μM) on IL-2 was significantly lower in COPD patients compared to NS (p=0.001). Dexamethasone significantly reduced IL-2, IL-10, IL-13, and IL-17 release from NS and COPD patients; this ranged between 57-94% for NS and 57-93% for COPD patients, with no differences between groups.

### Epithelial cell culture

As we had found LXRα and LXRβ immunostaining in bronchial epithelial cells of COPD patients and controls, we investigated the functional effects of LXR activation on cytokine production from a human bronchial epithelial cell line (BEAS-2B). Firstly, we confirmed that this cell line expressed LXRα and LXRβ (Additional file 10).

Stimulation of BEAS-2Bs with poly I:C (10 μg/ml), but not LPS (1 μg/ml), significantly increased the production of CXCL10 (p=0.01). Poly I:C stimulation was therefore used to investigate the anti-inflammatory effects of LXR activation. Pre-treatment of BEAS-2Bs with GW3965 did not significantly inhibit the production of CXCL10 (Additional file 10). In contrast, pre-treatment with dexamethasone significantly inhibited CXCL10 production by 89% (p=0.02).

## Discussion

We now summarise our findings; LXR gene and protein expression levels were similar in COPD macrophages compared to controls. GW3965 upregulated the expression of the known LXR target genes ABCA1 and ABCG1 in COPD alveolar macrophages, confirming the pharmacological activity of this drug on these cells. However, there was no effect on M2 gene expression. GW3965 had a modest inhibitory effect on the production of some cytokines including CCL5 and CXCL10, with the clearest effect in COPD macrophages observed on CXCL10 mRNA levels. There was also an increase in the production of the anti-inflammatory cytokine IL-10. However, LXR activation did not suppress the production of TNFα, IL-1β or CXCL8. It appears that LXR activation had modest anti-inflammatory effects on COPD alveolar macrophages; with notable effects on CXCL10 (suppression) and IL-10 (increased) production.

GW3965 inhibited CXCL10 and CCL5 secretion and increased IL-10 production in COPD and S alveolar macrophages, with the maximum changes being approximately 40% in both groups. Despite the similar numerical magnitude of effect in both groups, statistical significance was observed in S only. However, gene expression experiments demonstrated that GW3965 reduced CXCL10 expression in COPD macrophages. We suggest that the overall interpretation of these cytokine protein and gene expression data is that GW3965 has similar effects in both COPD and S alveolar macrophages, but that the sample size in the COPD protein experiments was insufficient to demonstrate statistical significance. Alternatively, the discordance between gene and protein data maybe due to post-transcriptional mechanisms which interfere with the efficient translation of the mRNA product to the mature protein.

We subsequently pooled the COPD and S protein data with interesting consequences; the effects of GW3965 became apparent at 1 μM as well as 10 μM for CCL5, highlighting the increased statistical power of this pooled analysis. Furthermore, the increased sample size allowed inhibitory effects on GM-CSF and IL-6 to become apparent. Nevertheless, the magnitude of inhibition achieved in this pooled analysis was 41% or lower, which was generally less than the corticosteroid dexamethasone. However, the suppressive effect of GW3965 on CXCL10 was at least equal to corticosteroid. Furthermore, GW3965 increased IL-10 production, while corticosteroid reduced the levels of this anti-inflammatory cytokine.

LXR and the glucocorticoid receptor (GR) are nuclear hormone receptors that are known to target subsets of the inflammatory genome [14]. Our findings indicate that GR activation has much greater efficacy than LXR activation on many pro-inflammatory cytokines released by COPD macrophages. However, LXR activation may still have some useful anti-inflammatory effects that are driven through CXCL10 inhibition and increased IL-10 production.

The effects of LXR activation are different in our study and the report of Birrel et al.; for example, we did not observe TNFα and CXCL8 suppression, although both studies demonstrated IL-6 suppression. The differences are hard to explain. Both studies obtained macrophages from surgical specimens. We clinically classified the patients as COPD or controls, but did not observe a difference between groups; differences between patients therefore cannot explain the lack of effect that we have observed. We demonstrated that GW3965 activated LXR; this was verified by ABCA1 and ABCG1 gene expression upregulation. We speculate that the effect of LXR on the production of some macrophage derived cytokines is at best modest; Birrel et al. showed inhibition that was often below 50%. Such modest effects may not be reproducible.

CXCL10 levels are increased in the lungs of COPD patients [30]; this chemokine plays a role in T lymphocyte chemotaxis through binding to CXCR3. The number of CD8 cells and the expression of CXCR3 are increased in the lungs of COPD patients [30], suggesting a prominent role for CXCL10 – CXCR3 interactions in the control of lymphocyte chemotaxis in COPD. Furthermore, the number of lymphoid follicles in the lungs increases with COPD severity [31]; these are organised structures that control antigen presentation and adaptive immune function. CXCL10 is involved in the organisation of these follicles [32]. LXR agonists may have a potential therapeutic role in COPD through inhibition of the production of this chemokine.

CXCL10 is an interferon inducible protein whose transcription is regulated by STAT1 in response to interferon gamma (IFN-γ) [33] and LPS [26] exposure. Li *et. al* reported that the endogenous LXR agonist, 22-(R)-hydroxycholesterol, reduces IFN-γ stimulated STAT1 phosphorylation in THP-1 macrophages [27]. In contrast, we found GW3965 did not reduce STAT1 phosphorylation in alveolar macrophages. These differences may be due to ligand specificities, as Li *et. al* also found that the LXR agonist T01317 did not reduce STAT1 phosphorylation. In the same study, T01317 and 22-(R)-hydroxycholesterol were shown to attenuate STAT1 DNA binding [27]. The same observations have also been demonstrated in rat brain astrocytes [17]. We hypothesise that this is the mechanism by which LXR causes inhibition of LPS stimulated CXCL10 in alveolar macrophages.

COPD macrophages are skewed towards the alternative activation phenotype [19]. The phenotypic activity of macrophages is probably under dynamic control of extracellular signals. This regulation involves the cholesterol transporter ABCA1 in murine macrophages [21]. We have shown that LXR activation of ABCA1 does not promote the transcription of M2 genes in COPD macrophages, thus ruling out a potentially therapeutic role for LXR agonists in altering macrophage phenotype in COPD.

The gene expression levels of LXRα and LXRβ in whole lung tissue of COPD patients and S were increased compared to NS, indicating that chronic cigarette smoke exposure upregulates LXR gene expression. There was also an increase in the number of LXR immunoreactive bronchial and alveolar epithelial cells in COPD patients, with evidence for higher expression compared to both S and NS, suggesting that the development of COPD is associated with an upregulation of LXR protein expression in these specific cell types.

The whole lung gene expression levels did not match the protein expression data for individual cell types, as the protein data showed more evidence for upregulation of LXR expression due to COPD itself, rather than cigarette smoking alone. Whole lung gene expression takes into account all cell types, which may have variations in LXR regulation. Furthermore, it is not possible to control for the proportion of different cell types in whole lung samples, and the presence of inflammation and emphysema in COPD samples will alter the proportion of cell types present compared to controls. The gene and protein expression data from alveolar macrophages were similar, showing no differences between groups; this demonstrates good agreement between gene and protein expression when cell types are matched. Protein expression is ultimately more relevant for physiological function, and we suggest that our data for protein is more relevant, showing that COPD patients have increased LXR expression in bronchial and alveolar epithelial cells.

LXR regulates its own expression; LXR activation increases LXR gene expression in human macrophages [34,35]. LXR activation in the lungs of COPD patients could be through the endogenous ligands 25- and 27-hydroxycholesterol, both of which are increased in the induced sputum of COPD patients [36,37]. The expression levels of the hydroxylases responsible for the production of these oxysterols are also increased in the lung tissue of COPD patients [36,37]. Abnormal lipid metabolism could therefore be the cause of increased LXR expression in the lungs of COPD patients, with LXR acting in these circumstances to promote cholesterol efflux [8,20]. LXR transcription is also regulated by PPAR (peroxisome proliferator-activated receptor) γ [38,39] and by cigarette smoke directly [40]. These may also influence LXR expression and therefore LXR dependent cholesterol efflux in the lungs of COPD patients.

This is the first study to compare the effects of LXR activation on lymphocyte derived cytokines from COPD patients and NS PBMCs. GW3965 inhibited IL-2 and IL-17 production, with a reduced effect observed in COPD patients. Walcher *et. al* also showed that LXR activation using T01317 reduced anti-CD3/28 stimulated IL-2 release from NS PBMCs by a similar magnitude to our results; approximately 20%. The anti-inflammatory effects of LXR activation were much lower than dexamethasone in the current study, and were also more cytokine selective as only IL-2 and IL-17 production were inhibited. It is known that nuclear hormone receptors only target a proportion of the inflammatory genome [14], and it seems that LXR activation causes a restricted anti-inflammatory effect compared to corticosteroids in lymphocytes. The number of lymphocytes in the lungs of COPD patients are increased [31], and these cells release a variety of cytokines [41,42]. The restricted nature of the anti-inflammatory activity of LXR on selected cytokines in lymphocytes, coupled with the reduced effect size compared to corticosteroids, makes it unlikely that the *in-vitro* anti-inflammatory effects reported here would translate into clinically meaningful benefits in COPD patients. Furthermore, the reason for the lower efficacy of GW3965 in COPD patients compared to controls is unclear, but casts additional doubt on whether LXR activation would produce meaningful effects on lymphocyte activation in COPD.

Although GW3965 reduced LPS stimulated CXCL10 production in alveolar macrophages, we did not observe inhibition of poly I:C stimulated CXCL10 production in bronchial epithelial cells. These differences in findings are likely to be due to differences between cell types and/or the activating stimulus used. Similarly, the effect of corticosteroid on CXCL10 release from bronchial epithelial cells was greater than that observed in alveolar macrophages, which again may be attributed to cell type and/or stimulus. Nevertheless, the increased expression of LXR in bronchial epithelial cells in COPD patients suggests a role for this protein in the pathophysiology of COPD. This may be elucidated through further studies investigating the effects of LXR on the transactivation and transrepression of epithelial genes. There is data showing that LXR activation in human monocytes increases TNFα mRNA levels and intracellular protein accumulation prior to release [43]. LXR activation has also been shown to worsen disease progression in murine models of asthma [44] and arthritis [45]. Furthermore, LXR activation can increase TLR4 gene expression which may lead to an exaggerated response to LPS [29]. We did not observe any increase in TLR4 expression, or any increase in cytokine release from alveolar macrophages caused by LXR activation, suggesting that LXR activation in this cell type does not cause pro-inflammatory effects.

We have previously demonstrated that corticosteroids have a limited inhibitory effect on the production of some cytokines from COPD alveolar macrophages [22,46,47]. The effect of dexamethasone in the current study is similar to our previous observations, including the finding of a modest effect on CXCL10 production [47].

## Conclusion

Previous reports have showed that LXR agonists suppress the production of some cytokines from macrophages [13,18], leading us to evaluate the potential of LXR activation in COPD macrophages. We observed that LXR activation caused only modest anti-inflammatory effects on selected cytokines released from COPD alveolar macrophages. The most interesting findings were that CXCL10 production was suppressed and that IL-10 production was increased. LXR agonists are being developed as a potential treatment for cardiovascular disease [11]. Perhaps the most appropriate clinical avenue for the development of LXR agonists in COPD would be for those patients with concurrent cardiovascular disease, as a dual benefit on plaque formation coupled with anti-inflammatory effects in the lung could be observed.

## Competing interests

AH, SL, JP, and DR have no conflicts of interest to disclose. BM and KS are employees of GlaxoSmithKline. DS has received sponsorship to attend international meetings, honoraria for lecturing or attending advisory boards, and research grants from various pharmaceutical companies including AstraZeneca, Boehringer Ingelheim, Chiesi, GlaxoSmithKline, Almirall, Forest, Pfizer, UCB, Novartis, and Cipla.

## Authors’ contributions

AH: carried out main body of lab work, data analysis and manuscript composition. SL: data analysis and manuscript composition. JP: involved in immunohistochemistry, data analysis and manuscript composition. BM: involved in luminex, data analysis and manuscript composition. KS: data analysis and manuscript composition. DR: involved in study design, data analysis and manuscript composition, DS: involved in study design, data analysis and senior contribution to manuscript composition. All authors read and approved the final manuscript.

## Supplementary Material

Additional file 1 **Subject demographics for peripheral blood donors.** Data shown are mean (sd). NS: non-smokers, FEV_1_: forced expiratory volume in 1s, FVC: forced vital capacity, ICS: inhaled corticosteroid. Click here for file

Additional file 2 Materials and methods. Click here for file

Additional file 3 **Representative images of LXRα and LXRβ expression in lymphocytic aggregates.** The distribution of LXRα (A, C, and E) and LXRβ (B, D, and F) within lymphocytic aggregates present in lung sections of non-smoking controls (NS) (A-B), smoking controls (S) (C-D), and COPD patients (E-F). Click here for file

Additional file 4 **The effect of GW3965 on ABCA1 and ABCG1 mRNA expression in alveolar macrophages.** Macrophages from smoking controls (A and C) (n=8) and COPD patients (B and D) (n=8) were treated with or without GW3965 (1 μM) for 4, 24 and 48 h. RNA was extracted for PCR analysis of ABCA1 (A and B) and ABCG1 (C and D) mRNA expression. Data shown are median ± range of fold increase of mRNA expression above time matched controls. * and ** = significant difference compared to time matched controls (p<0.05 and p<0.01 respectively). Click here for file

Additional file 5 **The effect of LPS on cytokine and chemokine production from alveolar macrophages.** Data shown are mean (sd) or median ± range from 8 S and 7 COPD patients. *, **, and *** = significant difference from unstimulated control (p<0.05, p<0.01, and p<0.001 respectively). S: smokers. Click here for file

Additional file 6 **The expression of CXCL10 mRNA in LPS stimulated alveolar macrophages.** Macrophages from smoking controls (n=8) (A) and COPD patients (n=8) (B) were stimulated with LPS (1 μg/ml) for 6 or 24 h prior to RNA extraction and PCR analysis for CXCL10. Data shown are median ± range. * = significant difference compared to time matched control (p<0.05). Click here for file

Additional file 7 **The effect of GW3965 on the phosphorylation of STAT1 (727).** Macrophages from smoking controls (n=3) (A) and COPD patients (n=3) (B) were treated with vehicle control (DMSO 0.05%) or GW3965 (1 μM or 10 μM) for 1 h prior to stimulation with LPS (1 μg/ml) for 1 h. Macrophages were then lysed and samples were analysed for phosphorylated STAT1 (727) by western blot. All blots were analysed by densitometry and any changes were relative to the loading control β-actin. Data shown are mean ± SEM with representative blots below. * = significant difference compared to unstimulated control (p<0.05). Click here for file

Additional file 8 **The effect of GW3965 on macrophage polarisation.** Alveolar macrophages from smoking controls (A, C, E, and G) (n=8 apart from at 10 μM n=5) and COPD patients (B, D, F, and H) (n=9 apart from at 10 μM n=3) were treated with or without GW3965 (1 μM and 10 μM) for 4, 24 and 48 h. RNA was extracted for PCR analysis of HO-1 (A and B), CD36 (C and D), MR (E and F) and TLR4 (G and H) mRNA expression. Data shown are mean ± SEM of fold increase of mRNA expression above time matched controls. Click here for file

Additional file 9 **The effect of anti-CD2/3/28 stimulation on cytokine production from PBMCs.** Data shown are mean (sd) or median ± range from 10 NS and 10 COPD patients. * = significant induction of mediator compared to unstimulated control (p<0.001). NS: non-smokers. Click here for file

Additional file 10 **The effect of GW3965 on the production of CXCL10 from poly I:C stimulated BEAS-2Bs.** Immunocytochemical staining confirmed the presence of LXRα (A) and LXRβ (B) in BEAS-2Bs. Omission of the primary antibodies displayed no immunoreactivity for LXRα (C) and LXRβ (D). (E) BEAS-2Bs (n=3) were pre-treated with vehicle (DMSO 0.05%) (white bars), GW3965 (1 μM or 10 μM) (light grey bars), or dexamethasone (1 μM) (dark grey bars) for 1 h prior to stimulation with poly I:C (10 μg/ml) for 24 h. Culture supernatants were analysed for CXCL10. Data shown are mean ± SEM where * = significant reduction of CXCL10 below vehicle control (p<0.05). Click here for file

## Footnotes

This work was kindly funded by GlaxoSmithKline and the BBSRC.
