# Supplementary material for: The role of the liver X receptor in chronic obstructive pulmonary disease
Source: Respir Res. 2013 Oct 12;14(1):106. doi: 10.1186/1465-9921-14-106 (PMC3852990; doi:10.1186/1465-9921-14-106)
Supplement: Additional file 1 — Subject demographics for peripheral blood donors. Data shown are mean (sd). NS: non-smokers, FEV1: forced expiratory volume in 1s, FVC: forced vital capacity, ICS: inhaled corticosteroid. [file 1465-9921-14-106-S1.docx]

**Additional File 1**

|  | **NS** | **COPD** |
| --- | --- | --- |
| **n** | 10 | 10 |
| **Age (yrs)** | 36.4 (17.1) | 66.1 (9.4) |
| **Sex (M/F)** | 5/5 | 6/4 |
| **FEV_1_ (L)** | 4.5 (0.9) | 1.4 (0.5) |
| **FEV_1_ % Predicted** | 118.4 (12.0) | 49.4 (18.5) |
| **FVC (L)** | 5.4 (0.9) | 3.2 (0.7) |
| **FEV_1_/FVC Ratio (%)** | 83.4 (7.0) | 44.5 (10.6) |
| **Pack Year History** | 0 | 57.4 (33.0) |
| **Current smoker (%)** | 0 | 60 |
| **ICS users** | 0 | 6 |

**Subject demographics for peripheral blood donors.** Data shown are mean (sd). NS: non-smokers, FEV_1_: forced expiratory volume in 1s, FVC: forced vital capacity, ICS: inhaled corticosteroid.
