# Supplementary material for: The role of the liver X receptor in chronic obstructive pulmonary disease
Source: Respir Res. 2013 Oct 12;14(1):106. doi: 10.1186/1465-9921-14-106 (PMC3852990; doi:10.1186/1465-9921-14-106)
Supplement: Additional file 2 — Materials and methods. [file 1465-9921-14-106-S2.docx]

**Additional File 2**

**MATERIALS AND METHODS**

**Tissue Sampling and Processing**

Tissue blocks were obtained from 10 NS, 10 S, and 10 currently smoking COPD patients as far distal to the tumour as possible then formalin fixed and paraffin embedded. Tissue sections were cut to 4 μm thick and mounted onto polysine coated glass slides. For each patient, sections were labelled using either monoclonal mouse anti-human LXRα (ligand binding domain, clone PPZ0412, R&D Systems, Abingdon, UK) or polyclonal goat anti-human LXRβ (clone S-16: sc-34343, Santa Cruz Biotechnology Inc., Santa Cruz, CA) primary antibodies. Sections were chosen based on the presence of small airways identified through haematoxylin and eosin archival staining.

Heat induced epitope retrieval was conducted by microwaving for 20 min at 800 W in a 1 mM trisodium citrate buffer pH6 for sections to be labelled with LXRα antibody and in a Tris-EDTA buffer pH8 (10 mM Tris Base, 1 mM EDTA, 0.5% Tween20) for sections to be labelled with LXRβ antibody. Primary antibody diluted in 1.5% normal serum (Vector Labs, Peterborough, UK) was applied overnight at 4^o^C. Endogenous peroxidase was quenched by incubating sections in 3% H_2_O_2_ in methanol for 30 minutes at room temperature. LXRα was detected using biotinylated horse anti-mouse immunoglobulin (Ig)G secondary antibody (Vector Labs) and LXRβ using biotinylated rabbit anti-goat immunoglobulin (Ig)G secondary antibody (Vector Labs), in conjunction with an avidin-biotin peroxidase complex (Vector Labs) and diaminobenzidine substrate. Sections were counterstained with Meyer’s haematoxylin (Sigma, Poole, UK). Omission of primary antibody from staining protocol and substitution of primary antibody with an isotype control antibody for LXRα (Dako UK Ltd, Cambridgeshire, UK) and LXRβ (Vector Labs) were used as negative controls.

**Image Capture and Morphometric Analysis**

The number of LXRα^+^ and LXRβ^+^ cells were calculated within the alveolar walls, and the epithelial and subepithelial layers (within an area of 100 µm) of small airways; airways devoid of cartilage and glandular tissue and with an internal perimeter of <6 mm. The number of peripheral macrophages displaying immunoreactivity was also quantified. Digital micrographs were obtained using a Nikon Eclipse 80i microscope (Nikon UK Ltd, Surrey, UK) equipped with a QImaging digital camera (Media Cybernetics, Marlow UK) and quantified using ImagePro Plus 5.1 software (Media Cybernetics). Cell counts were calculated and standardised to the number of immunoreactive cells per mm^-1^ (alveolar wall and airway epithelial layer), or cells per mm^-2^ (airway subepithelial layer), or to a total count of 200 peripheral macrophages obtained from non-consecutive frames.

**Whole Lung Tissue Preparation**

Resected lung tissue from 10 NS, 10 S, and 10 currently smoking COPD patients as far distal to the tumour as possible was snap frozen and cut into 30 µM sections using the Bright OTF 5000 Cryostat and then lysed with Trizol (Invitrogen, Paisley, UK) prior to RNA extraction and real time PCR analysis for LXRα and LXRβ expression.

**Alveolar Macrophage Culture**

***The Expression of LXRα and LXRβ***

Macrophages from 10 NS, 10, S, and 10 currently smoking COPD patients were lysed with Trizol prior to RNA extraction and real time PCR analysis for LXRα and LXRβ expression.

***Inflammatory Mediator Production***

Macrophages were cultured with GW3965 (1 or 10 µM), dexamethasone (1 µM) (Sigma-Aldrich), or control for 1 h followed by LPS (1 μg/ml, *Escherichia Coli* B6-026, Sigma-Aldrich) stimulation. Supernatants were removed at 24 h and analysed for IL-6 and CXCL8 by ELISA or GM-CSF, IL-1β, IL-10, CCL5, CXCL10, and TNF-α by multiplex. At 6 and 24 h the cells were lysed with Trizol prior to RNA extraction and real time PCR analysis for CXCL10 expression levels

**PBMC Culture**

Peripheral blood was obtained by venous extraction using heparin tubes prior to isolation of PBMCs. Blood was then layered onto Ficoll-Paque and centrifuged (400 g, 30 minutes, 4^o^C) to achieve density gradient separation. The mononuclear cells at the Ficoll interface were extracted, re-suspended in RPMI 1640 media supplemented with 10% foetal bovine serum (Invitrogen), 1% penicillin/streptomycin (Sigma-Aldrich), and 1% L-glutamine (Invitrogen) and washed (400 g, 10 minutes, 4^o^C). PBMC number was assessed using trypan blue exclusion and resuspended at a concentration of 2 x 10^6^ ml^-1^ in RPMI 1640 supplemented media. PBMCs were seeded onto round bottomed 96-well plates at a concentration of 2x10^5^ PBMCs per well. PBMCs were stimulated with the T-cell activation/expansion kit (Miltenyi Biotech Ltd, Surrey, UK) according to manufacturer’s instructions.

**Epithelial Cell Culture**

BEAS-2Bs are a cell line derived from human bronchial epithelium transformed by an adenovirus 12-SV-40 virus (1). Cells were suspended in RMPI supplemented with 10% foetal bovine serum, 1% penicillin/streptomycin, 1% L-glutamine, 1% HEPES (Life Technologies, Warrington, UK), and 1% sodium bicarbonate (Life Technologies). Cells were seeded onto T75cm^2^ culture flasks and incubated in a 5% CO_2_ humidified atmosphere at 37^o^C until they reached 80% confluence. Once at confluence, cells were washed with PBS and detached from the flask using 0.25% trypsin-EDTA (Life Technologies). The reaction was terminated by the addition of RPMI supplemented media and the suspension was centrifuged (400 g, 10 minutes, 4^o^C). Cell number was assessed using trypan blue exclusion and resuspended at a concentration of 1 x 10^6^ ml^-1^ in RMPI supplemented media. Cells were sub-cultured in T75cm^2^ culture flasks or seeded onto flat bottomed 96-well plates at a concentration of 20 x 10^4^ cells per well for experiments measuring supernatant cytokine levels. Cells were also seeded onto 8-well glass chamber slides (Thermo Scientific, Surrey, UK) at a concentration of 20 x 10^4^ cells per well for immunocytochemical staining of LXRα and LXRβ and allowed to reach 80% confluence in a 5% CO_2_ humidified atmosphere at 37^o^C.

**Immunocytochemical Staining of LXRα and LXRβ in BEAS-2Bs**

Primary antibody diluted in 1.5% normal serum was applied overnight at 4^o^C. Endogenous peroxidase was quenched by incubating in 0.6% H_2_O_2_ in methanol for 30 minutes at room temperature. LXRα was detected using biotinylated horse anti-mouse immunoglobulin (Ig)G secondary antibody (Vector Labs) and LXRβ using biotinylated rabbit anti-goat immunoglobulin (Ig)G secondary antibody (Vector Labs), in conjunction with an avidin-biotin peroxidase complex and NovaRed peroxidase (Vector Labs). Cells were counterstained with Meyer’s haematoxylin.

**Cytokine and Chemokine Analysis**

Cytokine and chemokine levels in the supernatants from the cell culture experiments were analysed by: ELISA (R&D Systems, Abbingdon, UK) to quantify IL-6, CXCL8, and IL-2; Fluorokine MAP multiplex kits (R&D Systems) to quantify GM-CSF, IL-1β, IL-10, CCL5, CXCL10, TNFα, IL-13, and IL-17. All analysis techniques were used according to manufacturer’s instructions.

**RNA Extraction and PCR**

Total RNA was purified from cell lysates using RNeasy kits (Qiagen, Crawley, UK) according to manufacturer’s instructions. DNA contamination was prevented by on-column addition of DNase (Qiagen, Crawley, UK) according to manufacturer’s instructions. TaqMan reverse transcription- PCR (RT-PCR) was performed on 50 ng of RNA from lung macrophages using the Verso 2-step QRT-PCR kit (Thermo Scientific) according to manufacturer’s instructions. The resulting cDNA was reacted with ABsolute blue qPCR mix (Thermo Scientific) according to manufacturer’s instructions in 25 µl reactions containing premade ABI Taqman gene expression assays for either LXRα (Catalogue no: Hs00172885_m1), LXRβ (Catalogue no: Hs00173195_m1), ABCA1 (Catalogue no: Hs00194045_m1), ABCG1 (Catalogue no: Hs01555193_m1), TLR4 (Catalogue no: Hs01060206_m1), HO-1 (Catalogue no: Hs01110250_m1), CD36 (Catalogue no: Hs00354519_m1), MR (Catalogue no: Hs00267207_m1), or CXCL10 (Catalogue no: Hs00171042_m1 ), and the endogenous control was glyceraldehyde-3phosphate dehydrogenase (GAPDH) (Catalogue no: 4352934E) (all Applied Biosystems, Warrington, UK). Controls without RT-enzyme showed there was no genomic DNA amplification. Thermal cycling was carried out on a Stratagene MX3005P (Agilent Technologies, West Lothian, UK). Relative expression levels were determined using the ΔCt for the expression of LXRα/β and the ΔΔCt method for the expression of LXR dependant genes, with untreated cells as the calibrator.

**Protein Extraction and Western Blot**

Cells were lysed using RIPA buffer (10 mM Tris-HCl pH 7.4, 150 mM NaCl, 1mM EDTA, 1% Nonidet P-40) containing phosphatase (Sigma Aldrich, Dorset, UK) and protease inhibitors (Calbiochem, Nottingham, UK). Cell lysates diluted in sample buffer (62.5 mM Tris-HCl pH 6.95, 10% glycerol, 1% SDS, 1% β-mercaptoethanol, and 0.01% bromphenol blue) were sonicated for 10min at 4^o^C in a sonicating water bath and then boiled at 90^o^C for 5 minutes. The samples were then loaded and electrophoresed on SDS polyacrylamide gels (10%) at 150 V in 1x tris-glycine SDS (TGS) running buffer. The Precision Plus Protein Kaleidoscope Standard (Bio-Rad Laboratories Ltd, Hampshire, UK) was included as the molecular weight marker. Separated proteins were transferred to Hy-bond ECL membranes (Whatman, Kent, UK) at 400 mA in a 1x TGS, 20% methanol transfer buffer for 1 h. Membranes were incubated with blocking buffer (1% dried milk, 150mM NaCl containing 0.1% Tween 20) for 1hr at room temperature and then incubated with primary antibodies (diluted in blocking buffer) at 4 °C overnight. After washing in Tris-buffered saline containing 0.1% Tween 20 (TBS/Tween 20), the membranes were incubated for 60 minutes with a peroxidase-conjugated secondary antibody (diluted in wash buffer), washed again, and the antibody-labelled proteins were visualized by enhanced chemiluminescence (Amersham Biosciences, Buckinghamshire, UK). Densitometric analysis was performed by normalising band density to that for β-actin using Quantity One v4.6.1 software (Bio-Rad, Hertfordshire, UK).

The following antibodies were used; rabbit anti-phospho-STAT1 (Ser727) (Cell Signalling, Hitchin, UK), rabbit anti-βactin (Abcam, Cambridge, UK), and horseradish peroxidase-conjugated goat anti-rabbit (Cell Signalling).

**References**

1. American-Type-Culture-Collection. Available from: <http://www.lgcstandards-atcc.org/en.aspx>.
