# Supplementary material for: The role of the liver X receptor in chronic obstructive pulmonary disease
Source: Respir Res. 2013 Oct 12;14(1):106. doi: 10.1186/1465-9921-14-106 (PMC3852990; doi:10.1186/1465-9921-14-106)
Supplement: Additional file 3 — Representative images of LXRα and LXRβ expression in lymphocytic aggregates. The distribution of LXRα (A, C, and E) and LXRβ (B, D, and F) within lymphocytic aggregates present in lung sections of non-smoking controls (NS) (A-B), smoking controls (S) (C-D), and COPD patients (E-F). [file 1465-9921-14-106-S3.pptx]

## Slide 1
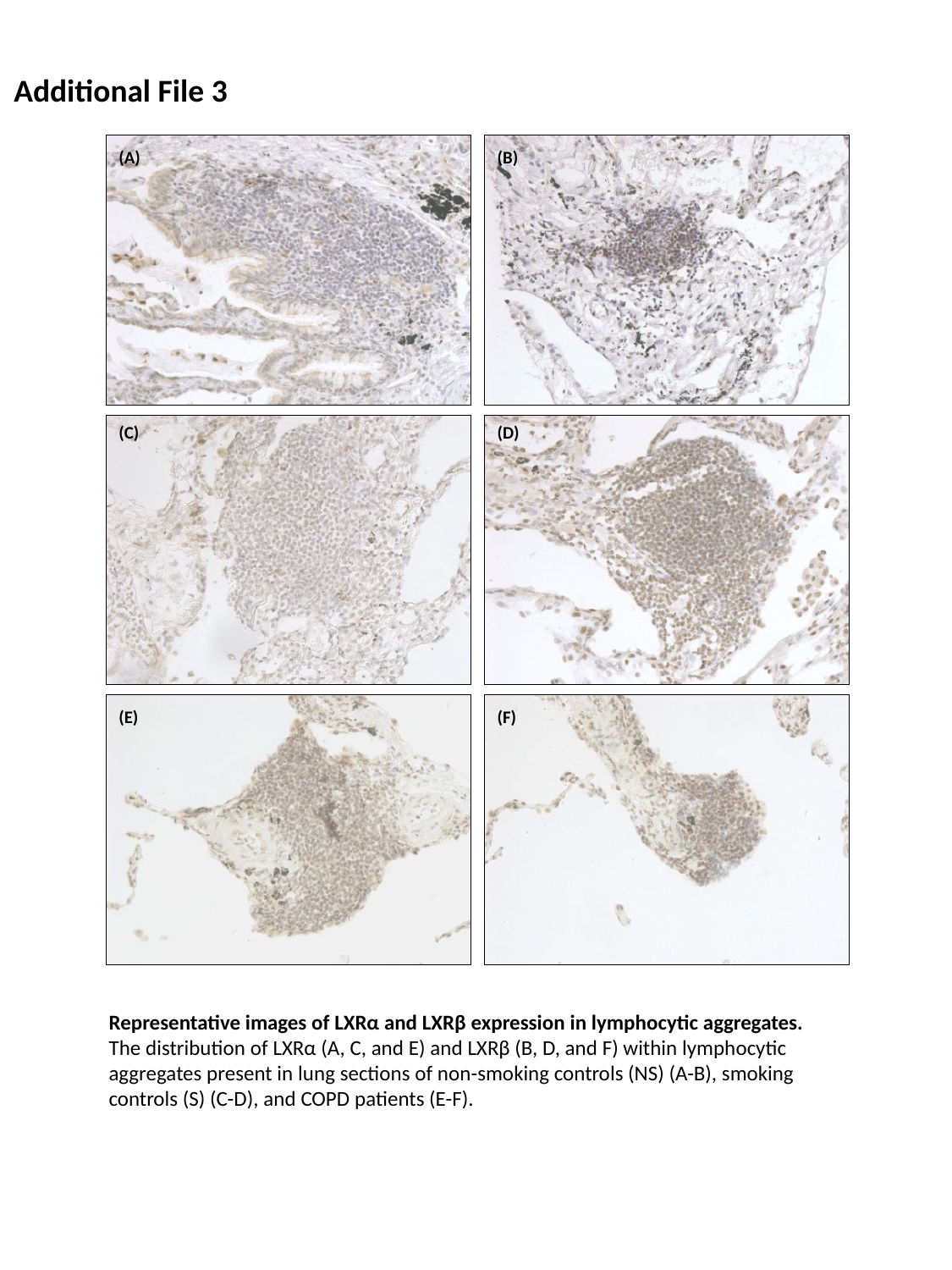

Additional File 3
(A)
(B)
(C)
(D)
(E)
(F)
Representative images of LXRα and LXRβ expression in lymphocytic aggregates.
The distribution of LXRα (A, C, and E) and LXRβ (B, D, and F) within lymphocytic aggregates present in lung sections of non-smoking controls (NS) (A-B), smoking controls (S) (C-D), and COPD patients (E-F).
