# Supplementary material for: The role of the liver X receptor in chronic obstructive pulmonary disease
Source: Respir Res. 2013 Oct 12;14(1):106. doi: 10.1186/1465-9921-14-106 (PMC3852990; doi:10.1186/1465-9921-14-106)
Supplement: Additional file 4 — The effect of GW3965 on ABCA1 and ABCG1 mRNA expression in alveolar macrophages. Macrophages from smoking controls (A and C) (n=8) and COPD patients (B and D) (n=8) were treated with or without GW3965 (1 μM) for 4, 24 and 48 h. RNA was extracted for PCR analysis of ABCA1 (A and B) and ABCG1 (C and D) mRNA expression. Data shown are median ± range of fold increase of mRNA expression above time matched controls. * and ** = significant difference compared to time matched controls (p<0.05 and p<0.01 respectively). [file 1465-9921-14-106-S4.pptx]

## Slide 1
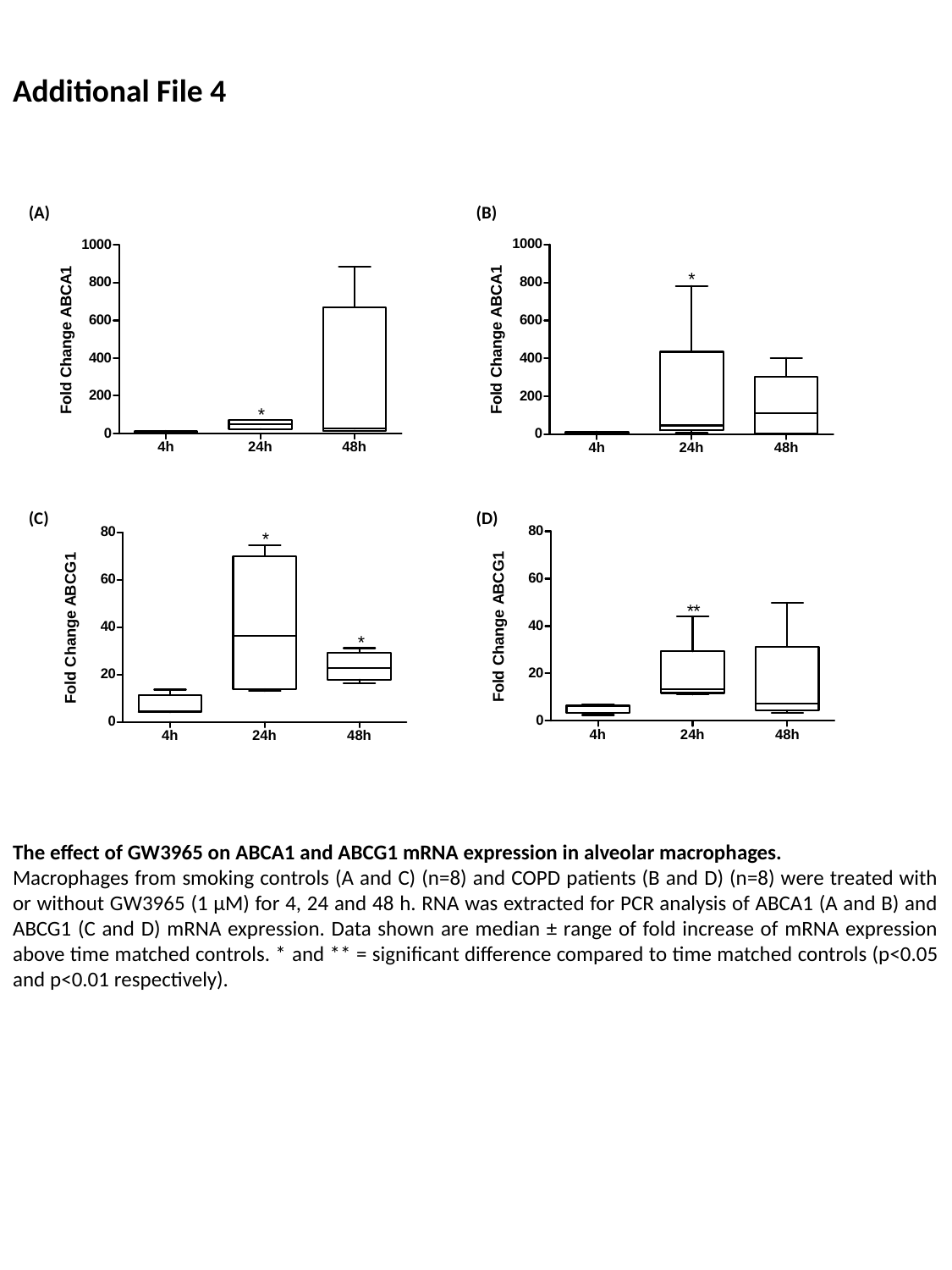

Additional File 4
(A)
(B)
(C)
(D)
The effect of GW3965 on ABCA1 and ABCG1 mRNA expression in alveolar macrophages.
Macrophages from smoking controls (A and C) (n=8) and COPD patients (B and D) (n=8) were treated with or without GW3965 (1 µM) for 4, 24 and 48 h. RNA was extracted for PCR analysis of ABCA1 (A and B) and ABCG1 (C and D) mRNA expression. Data shown are median ± range of fold increase of mRNA expression above time matched controls. * and ** = significant difference compared to time matched controls (p<0.05 and p<0.01 respectively).
