# Supplementary material for: The role of the liver X receptor in chronic obstructive pulmonary disease
Source: Respir Res. 2013 Oct 12;14(1):106. doi: 10.1186/1465-9921-14-106 (PMC3852990; doi:10.1186/1465-9921-14-106)
Supplement: Additional file 5 — The effect of LPS on cytokine and chemokine production from alveolar macrophages. Data shown are mean (sd) or median ± range from 8 S and 7 COPD patients. *, **, and *** = significant difference from unstimulated control (p<0.05, p<0.01, and p<0.001 respectively). S: smokers. [file 1465-9921-14-106-S5.docx]

**Additional File 5**

|  | **Unstimulated** | | **LPS stimulated (1 µg/ml)** | |
| --- | --- | --- | --- | --- |
| **Cytokine** | **S** | **COPD** | **S** | **COPD** |
| **CXCL10 (pg/ml)** | 270.5 (542.3) | 106.4  (257) | 2392.7 (1994.6)* | 1338.9 (1850.5) |
| **CCL5(pg/ml)** | 26.6  (19.4) | 11.9  (7.6) | 2058.6 (1268.3)** | 1587.8 (1526.5)* |
| **IL-10 (pg/ml)** | 2  (2.5) | 0.8  (0.6) | 180.5  (101)** | 182.6  (105.6)** |
| **GM-CSF (pg/ml)** | 4.2  (7.8) | 1.1  (2) | 494  (440.2)* | 474.8  (483.8)* |
| **IL-6 (pg/ml)** | 596.3 (947.6) | 838.9 (1596.5) | 83588.6  (75023.1)* | 116257.4  (119641.4)* |
| **CXCL8 (pg/ml)** | 13994.2  (10282.1) | 18191.7  (16766.2) | 441078.5  (210950.9)*** | 599063.9  (505213.5)* |
| **IL-1β (pg/ml)** | 3.8  (4.8) | 3.8  (4.8) | 201.3 ± 69.04-1930** | 229.8  (183.8)* |
| **TNFα (pg/ml)** | 20.5  (9.7) | 20.5  (9.7) | 9485.1  (4908)*** | 8929.5  (6769.6)* |

**The effect of LPS on cytokine and chemokine production from alveolar macrophages.** Data shown are mean (sd) or median ± range from 8 S and 7 COPD patients. *, **, and *** = significant difference from unstimulated control (p<0.05, p<0.01, and p<0.001 respectively). S: smokers
