# Supplementary material for: The role of the liver X receptor in chronic obstructive pulmonary disease
Source: Respir Res. 2013 Oct 12;14(1):106. doi: 10.1186/1465-9921-14-106 (PMC3852990; doi:10.1186/1465-9921-14-106)
Supplement: Additional file 6 — The expression of CXCL10 mRNA in LPS stimulated alveolar macrophages. Macrophages from smoking controls (n=8) (A) and COPD patients (n=8) (B) were stimulated with LPS (1 μg/ml) for 6 or 24 h prior to RNA extraction and PCR analysis for CXCL10. Data shown are median ± range. * = significant difference compared to time matched control (p<0.05). [file 1465-9921-14-106-S6.pptx]

## Slide 1
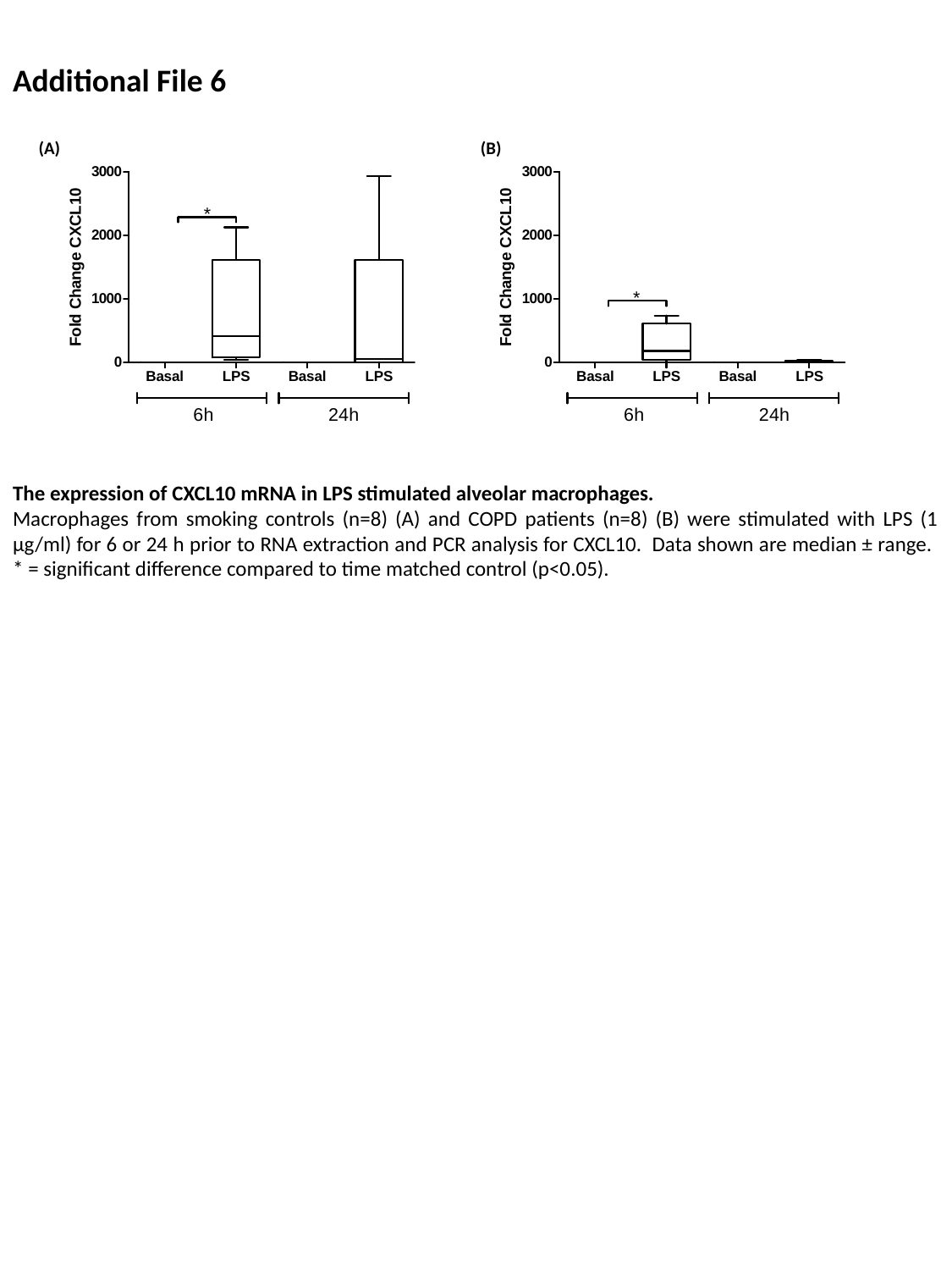

Additional File 6
(A)
(B)
The expression of CXCL10 mRNA in LPS stimulated alveolar macrophages.
Macrophages from smoking controls (n=8) (A) and COPD patients (n=8) (B) were stimulated with LPS (1 µg/ml) for 6 or 24 h prior to RNA extraction and PCR analysis for CXCL10. Data shown are median ± range. * = significant difference compared to time matched control (p<0.05).
