# Supplementary material for: The role of the liver X receptor in chronic obstructive pulmonary disease
Source: Respir Res. 2013 Oct 12;14(1):106. doi: 10.1186/1465-9921-14-106 (PMC3852990; doi:10.1186/1465-9921-14-106)
Supplement: Additional file 7 — The effect of GW3965 on the phosphorylation of STAT1 (727). Macrophages from smoking controls (n=3) (A) and COPD patients (n=3) (B) were treated with vehicle control (DMSO 0.05%) or GW3965 (1 μM or 10 μM) for 1 h prior to stimulation with LPS (1 μg/ml) for 1 h. Macrophages were then lysed and samples were analysed for phosphorylated STAT1 (727) by western blot. All blots were analysed by densitometry and any changes were relative to the loading control β-actin. Data shown are mean ± SEM with representative blots below. * = significant difference compared to unstimulated control (p<0.05). [file 1465-9921-14-106-S7.pptx]

## Slide 1
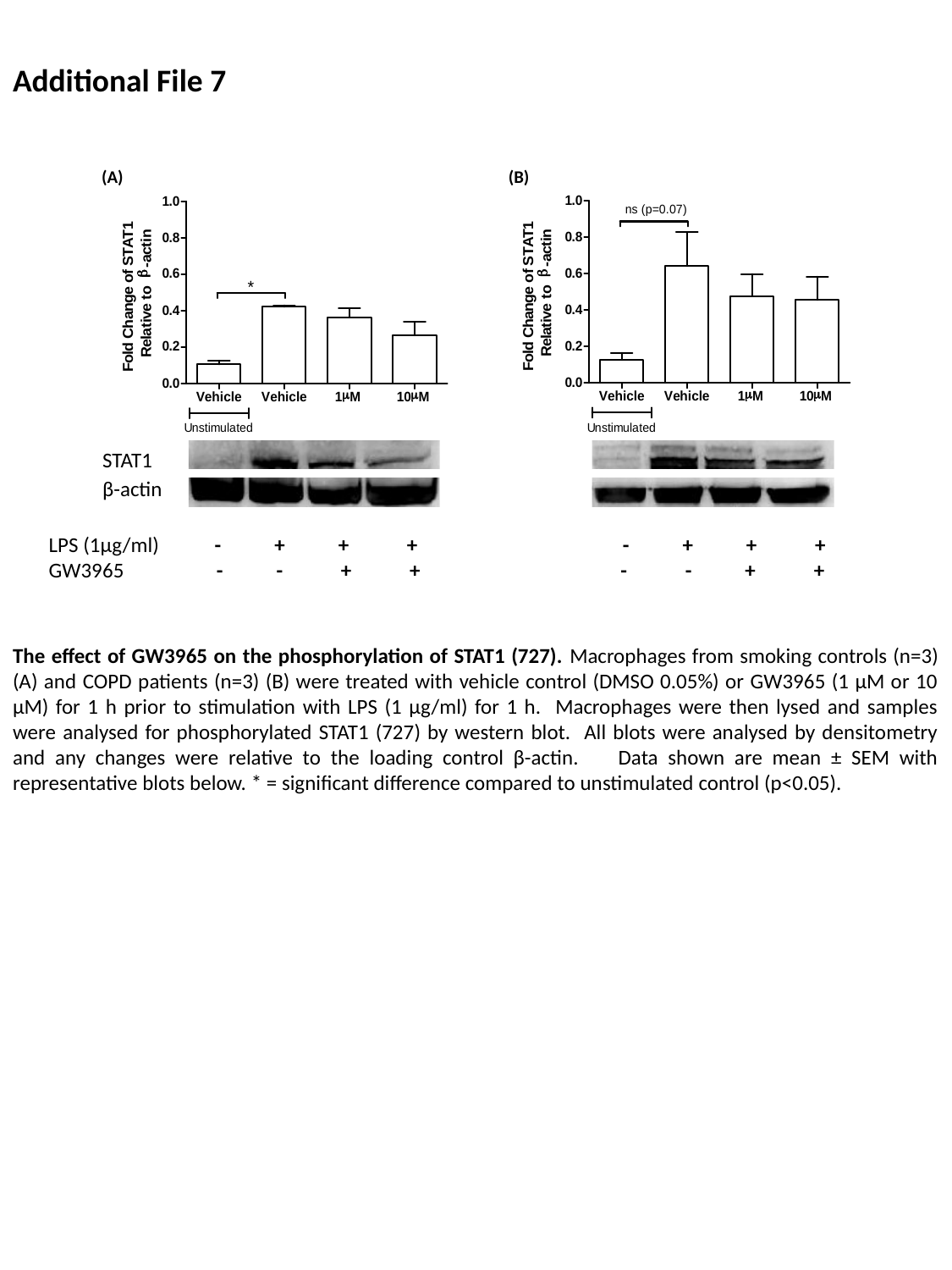

Additional File 7
(B)
(A)
STAT1
β-actin
LPS (1µg/ml)	 - + + + - + + +
GW3965 - - + + - - + +
The effect of GW3965 on the phosphorylation of STAT1 (727). Macrophages from smoking controls (n=3) (A) and COPD patients (n=3) (B) were treated with vehicle control (DMSO 0.05%) or GW3965 (1 µM or 10 µM) for 1 h prior to stimulation with LPS (1 µg/ml) for 1 h. Macrophages were then lysed and samples were analysed for phosphorylated STAT1 (727) by western blot. All blots were analysed by densitometry and any changes were relative to the loading control β-actin. Data shown are mean ± SEM with representative blots below. * = significant difference compared to unstimulated control (p<0.05).
