# Supplementary material for: The role of the liver X receptor in chronic obstructive pulmonary disease
Source: Respir Res. 2013 Oct 12;14(1):106. doi: 10.1186/1465-9921-14-106 (PMC3852990; doi:10.1186/1465-9921-14-106)
Supplement: Additional file 8 — The effect of GW3965 on macrophage polarisation. Alveolar macrophages from smoking controls (A, C, E, and G) (n=8 apart from at 10 μM n=5) and COPD patients (B, D, F, and H) (n=9 apart from at 10 μM n=3) were treated with or without GW3965 (1 μM and 10 μM) for 4, 24 and 48 h. RNA was extracted for PCR analysis of HO-1 (A and B), CD36 (C and D), MR (E and F) and TLR4 (G and H) mRNA expression. Data shown are mean ± SEM of fold increase of mRNA expression above time matched controls. [file 1465-9921-14-106-S8.pptx]

## Slide 1
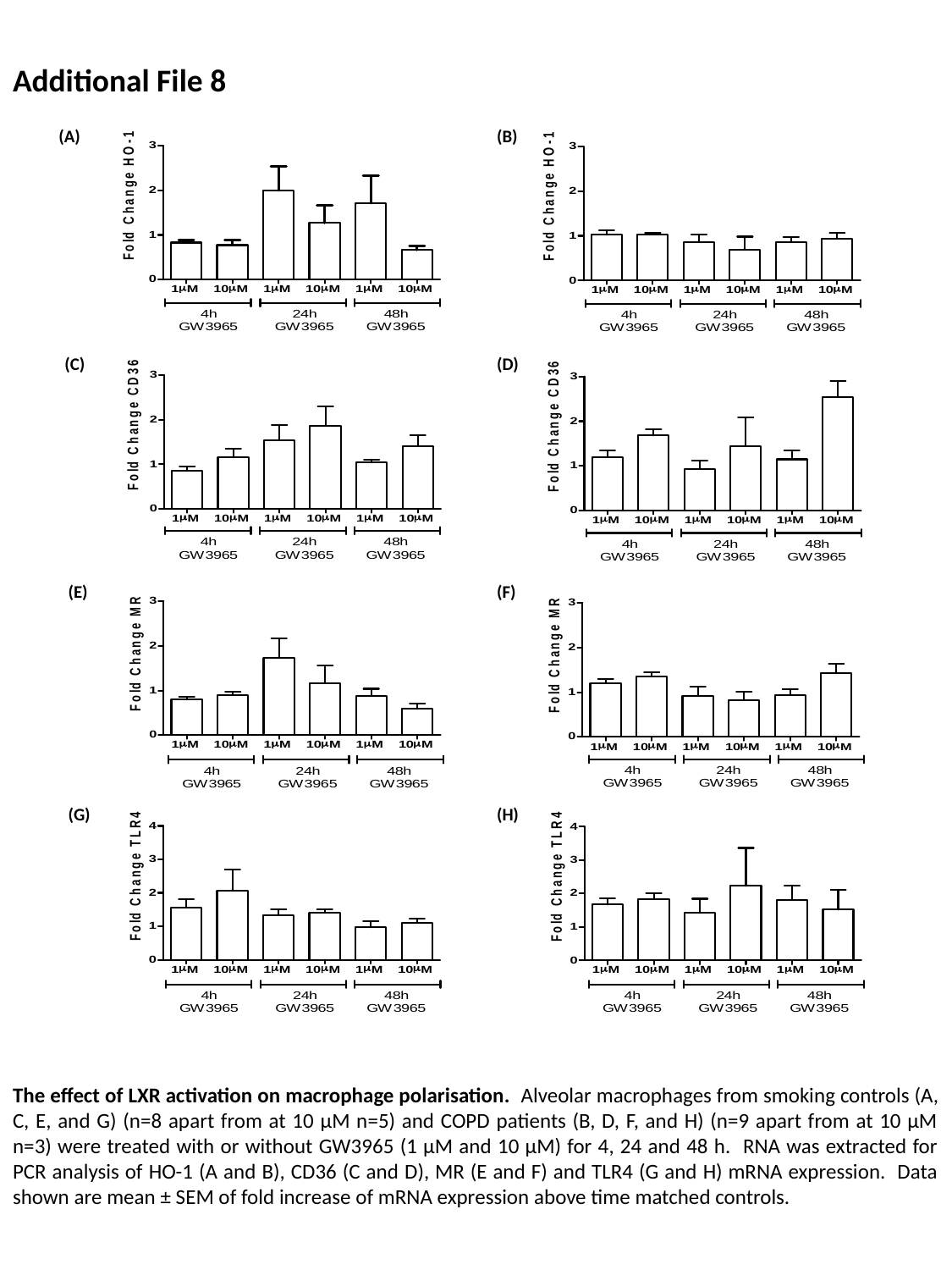

Additional File 8
(A)
(B)
(C)
(D)
(E)
(F)
(G)
(H)
The effect of LXR activation on macrophage polarisation. Alveolar macrophages from smoking controls (A, C, E, and G) (n=8 apart from at 10 µM n=5) and COPD patients (B, D, F, and H) (n=9 apart from at 10 µM n=3) were treated with or without GW3965 (1 µM and 10 µM) for 4, 24 and 48 h. RNA was extracted for PCR analysis of HO-1 (A and B), CD36 (C and D), MR (E and F) and TLR4 (G and H) mRNA expression. Data shown are mean ± SEM of fold increase of mRNA expression above time matched controls.
