# Supplementary material for: The role of the liver X receptor in chronic obstructive pulmonary disease
Source: Respir Res. 2013 Oct 12;14(1):106. doi: 10.1186/1465-9921-14-106 (PMC3852990; doi:10.1186/1465-9921-14-106)
Supplement: Additional file 9 — The effect of anti-CD2/3/28 stimulation on cytokine production from PBMCs. Data shown are mean (sd) or median ± range from 10 NS and 10 COPD patients. * = significant induction of mediator compared to unstimulated control (p<0.001). NS: non-smokers. [file 1465-9921-14-106-S9.docx]

**Additional File 9**

|  | **NS** | | **COPD** | |
| --- | --- | --- | --- | --- |
| **Cytokine** | **Unstimulated** | **Stimulated** | **Unstimulated** | **Stimulated** |
| **IL-2 (pg/ml)** | 6.6  (16.4) | 3369.2 (1049.0)* | 3.2  (6.9) | 2962.0 (1566.7)* |
| **IL-10 (pg/ml)** | 3.8  (4.3) | 821.3  (371.5)* | 6.3  (8.5) | 1188.7  (795.0)* |
| **IL-13 (pg/ml)** | 2.3  (4.2) | 337.4  (201.0)* | 0 ±  0-20.6 | 738.5 ± 283.6 – 4330.8* |
| **IL-17 (pg/ml)** | 1.4  (2.4) | 247.3  (148.5)* | 0.1 ±  0-7.3 | 86.7 ± 16.3 – 1101.1* |

**The effect of anti-CD2/3/28 stimulation on cytokine production from PBMCs.** Data shown are mean (sd) or median ± range from 10 NS and 10 COPD patients. * = significant induction of mediator compared to unstimulated control (p<0.001). NS: non-smokers.
