# Supplementary material for: The role of the liver X receptor in chronic obstructive pulmonary disease
Source: Respir Res. 2013 Oct 12;14(1):106. doi: 10.1186/1465-9921-14-106 (PMC3852990; doi:10.1186/1465-9921-14-106)
Supplement: Additional file 10 — The effect of GW3965 on the production of CXCL10 from poly I:C stimulated BEAS-2Bs. Immunocytochemical staining confirmed the presence of LXRα (A) and LXRβ (B) in BEAS-2Bs. Omission of the primary antibodies displayed no immunoreactivity for LXRα (C) and LXRβ (D). (E) BEAS-2Bs (n=3) were pre-treated with vehicle (DMSO 0.05%) (white bars), GW3965 (1 μM or 10 μM) (light grey bars), or dexamethasone (1 μM) (dark grey bars) for 1 h prior to stimulation with poly I:C (10 μg/ml) for 24 h. Culture supernatants were analysed for CXCL10. Data shown are mean ± SEM where * = significant reduction of CXCL10 below vehicle control (p<0.05). [file 1465-9921-14-106-S10.pptx]

## Slide 1
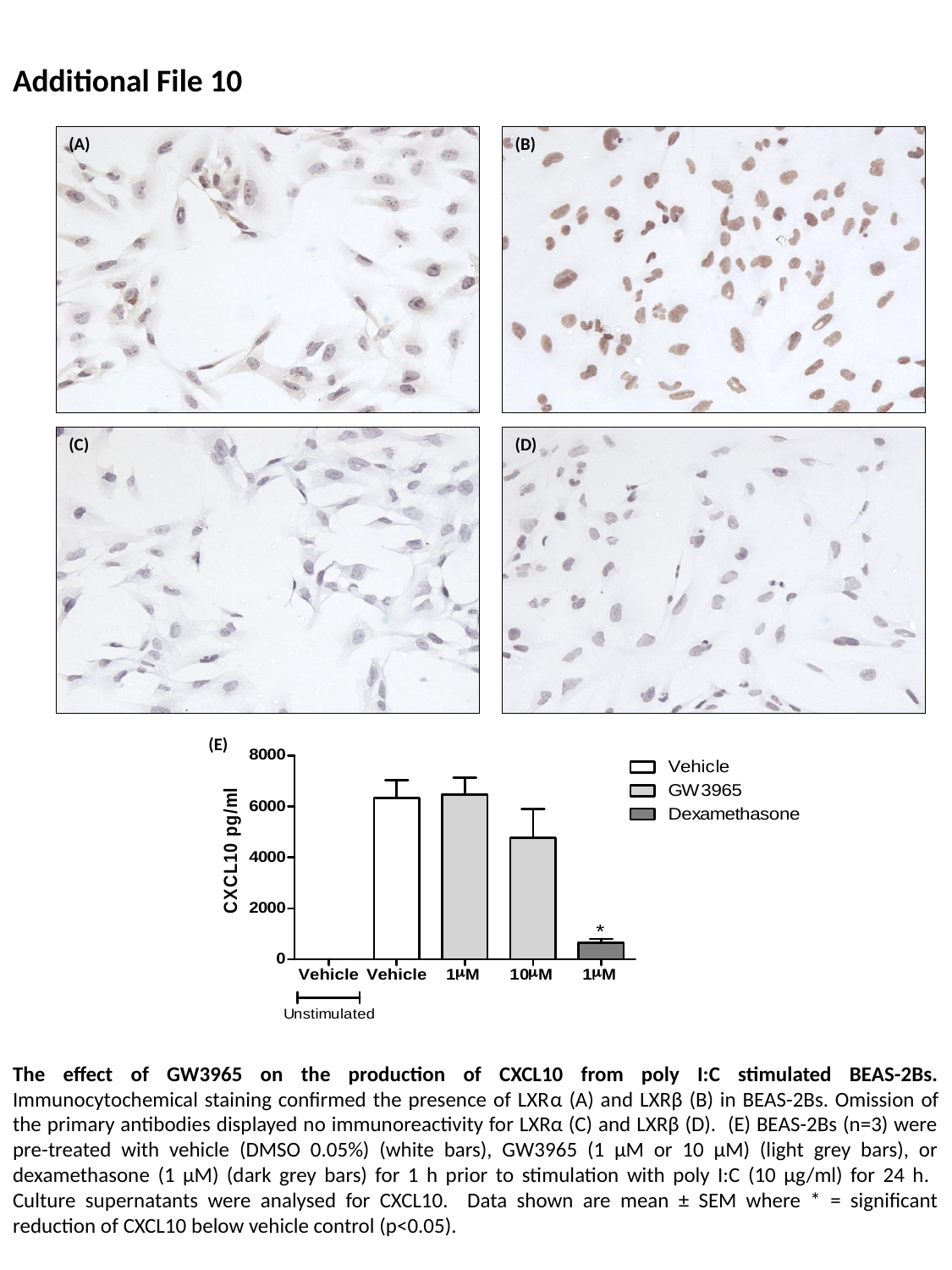

Additional File 10
(A)
(B)
(C)
(D)
(E)
The effect of GW3965 on the production of CXCL10 from poly I:C stimulated BEAS-2Bs. Immunocytochemical staining confirmed the presence of LXRα (A) and LXRβ (B) in BEAS-2Bs. Omission of the primary antibodies displayed no immunoreactivity for LXRα (C) and LXRβ (D). (E) BEAS-2Bs (n=3) were pre-treated with vehicle (DMSO 0.05%) (white bars), GW3965 (1 µM or 10 µM) (light grey bars), or dexamethasone (1 µM) (dark grey bars) for 1 h prior to stimulation with poly I:C (10 µg/ml) for 24 h. Culture supernatants were analysed for CXCL10. Data shown are mean ± SEM where * = significant reduction of CXCL10 below vehicle control (p<0.05).
